# WORKING TOGETHER TO PROVIDE GERIATRIC CARE: INTERDISCIPLINARY TEAM CREATION AND EVALUATION IN CANADA

**DOI:** 10.1093/geroni/igad104.2512

**Published:** 2023-12-21

**Authors:** Jessica Strong, Rachel Kerzner

**Affiliations:** University of Prince Edward Island, Charlottetown, Prince Edward Island, Canada; University of Prince Edward Island, Charlottetown, Prince Edward Island, Canada

## Abstract

Prince Edward Island is Canada’s smallest province (pop. 160,000) and has one of the oldest populations in Canada. While there is a robust and growing geriatrics program through provincial healthcare, it consists exclusively of medical providers (i.e., geriatricians and nurse practitioners). An opportunity for formal interprofessional collaboration presented itself when the University of Prince Edward Island began a Doctor of Psychology (PsyD) program in 2019. Soon thereafter, we established an interdisciplinary team between the PsyD and the provincial geriatrics programs. The goals of the team included: 1) enhancing care for older adults on the island, 2) providing training for PsyD students, and 3) increasing interprofessional collaboration and learning. In an evaluation of the first year of the collaboration, we collected data on the types of referrals and characteristics of the patients referred (e.g., number of medications), and asked all providers on the team (N=4 geriatricians, N=9 nurse practitioners, and N=4 psychology student clinicians) to answer open ended questions on the highlights and challenges of establishing the team, the organization of formal team meetings, and provision of patient care. Of the 74 patients seen by psychology, reasons included cognitive or psychodiagnostic assessment (N=22, 30%), psychotherapy (N=20, 27%), caregiver support (N= 25, 34%), or multiple services (i.e., both assessment and intervention; n=7, 10%). Additional patient characteristics will be shared, as well as themes from provider survey responses. Our program has implications for other areas in rural North America, particularly training opportunities built into our team functioning.

